# Euglycemic Diabetic Ketoacidosis Due to SGLT2 Inhibitor in a Patient With Gitelman Syndrome: A Therapeutic Dilemma

**DOI:** 10.7759/cureus.19169

**Published:** 2021-10-31

**Authors:** Taha Ahmed, Hussain Karimi, Vishwajit Hegde, Samra Haroon Lodhi

**Affiliations:** 1 Internal Medicine, University of Kentucky College of Medicine, Lexington, USA; 2 Internal Medicine, Cleveland Clinic, Cleveland, USA; 3 Internal Medicine, University of Kentucky, Lexington, USA; 4 Internal Medicine, Mayo Hospital, Lahore, PAK; 5 Internal Medicine, King Edward Medical University, Lahore, PAK

**Keywords:** sglt2 inhibitor, sodium-glucose cotransporter-2 inhibitors, euglycemic diabetic ketoacidosis, gitelman syndrome, dilemma

## Abstract

Euglycemic diabetic ketoacidosis (DKA) is a rarely reported side effect of sodium-glucose cotransporter-2 inhibitor (SGLT2i) empagliflozin. A 51-year-old female with Gitelman syndrome and type 2 diabetes mellitus (DM) presented with abdominal pain after a recent hospitalization for acute pancreatitis. Her diabetes medications included metformin, pioglitazone, and empagliflozin. Diabetic ketoacidosis was suspected; however, glucose levels were below the cutoff for DKA; therefore, she was diagnosed with euglycemic DKA. Her pancreatitis workup was insignificant. Severe symptomatic hypokalemia despite aggressive repletion limited the management of DKA with insulin infusion therapy. As her ketonemia resolved, she was initiated on subcutaneous insulin with a small but acceptable decrease in potassium. The therapeutic dilemma of managing euglycemic DKA due to SGLT2i in a patient with Gitelman syndrome has not been previously described.

## Introduction

Euglycemic diabetic ketoacidosis (DKA) is a clinical syndrome characterized by normal blood glucose level (less than 250 mg/dL), severe metabolic acidosis, and ketonemia. Originally described in type 1 diabetes mellitus (DM), the pathophysiology involves low insulin to glucagon ratio in the presence of a restricted pool of plasma glucose [1]. Sodium-glucose cotransporter-2 inhibitors (SGLT2i) are novel antihyperglycemic agents that act by reducing the reabsorption of glucose in the proximal renal tubule. SGLT2i are gaining popularity because of their favorable effect on patients with atherosclerotic cardiovascular disease and heart failure [2]. This report describes a case of euglycemic DKA, which is a rare but not uncommon side effect of this drug class. Gitelman syndrome is a renal tubulopathy that is characterized by the inability of the distal convoluted tubules to reabsorb sodium. Hypokalemic metabolic alkalosis and significant hypomagnesemia with low urinary calcium excretion are the hallmarks of the disease. The disease is usually inherited in an autosomal recessive manner with the prevalence being estimated at 1 in 40,000 in the Caucasian population, making Gitelman syndrome one of the most common inherited renal tubular disorders [3]. We report a case of euglycemic ketoacidosis due to SGLT2i in a patient with Gitelman syndrome. This case demonstrates an approach to the management of ketoacidosis complicated by severe baseline hypokalemia. As SGLT2i gain widespread use, it is essential to be cognizant of this potential side effect.

## Case presentation

A 51-year-old female presented to the emergency department with recurrent abdominal pain. She described her pain as diffuse, continuous, sharp, radiating to her back, and associated with nausea. The patient’s past medical history is significant for Gitelman syndrome diagnosed 30 years ago with chronic hypokalemia and hypomagnesemia (on oral replacement therapy), type 2 diabetes mellitus diagnosed 15 years ago with latest HbA1c of 8.1 (currently managed with metformin 1000 mg twice daily, pioglitazone 15 mg daily, and empagliflozin 25 mg daily), and gastric bypass surgery 11 years ago. Empagliflozin was added to her antihyperglycemic regimen three months ago, and she was tolerating it well. Her family history was significant for Gitelman syndrome in her grandmother. She was recently hospitalized for diffuse abdominal pain radiating to the back, nausea, diarrhea, poor oral appetite, and weight loss. During workup, a magnetic resonance cholangiography revealed mild focal acute pancreatitis. Her lipase level was elevated at 113 U/L (normal range: 19-63 U/L). The patient was managed with intravenous hydration and analgesia. Her condition improved, and she was discharged within 72 hours (four days before the current hospital presentation) upon her insistence to leave the hospital against medical advice. On presentation, the patient was afebrile with tachycardia (heart rate: 110-130 beats per minute), hypotension (blood pressure: 100/62 mmHg), and respiratory rate of 18 breaths per minute. Her physical examination was significant for tenderness to palpation on the suprapubic region and left upper abdominal quadrant. An electrocardiogram showed normal sinus rhythm, and cardiac enzymes were insignificant.

A complete blood count showed hemoconcentration and leukocytosis. A complete metabolic profile and arterial blood gas analysis revealed anion gap metabolic acidosis, severe hypokalemia, and acute kidney injury. Elevated ketones were found in the blood and urine. Lipase levels were normal, and computed tomography of the abdomen and pelvis showed no evidence of acute pancreatitis. COVID-19 testing was negative (Table 1). Telemetry monitoring revealed multiple frequent premature ventricular contractions.

**Table 1:** Peak daily values of the laboratory results

Laboratory investigation	Normal value	Day 1	Day 2	Day 3	Day 4	Day 5
Venous blood gas analysis (pH)	7.32–7.43	7.05		7.44		
PCO2 (mmHg)	37–52	24		31		
PO2 (mmHg)	25–40	34		41		
Bicarbonate (mmol/L)	22–26	7		21		
Lactate (mmol/L)	0.5–2.2	3.4	2.6	1.9		
Chemistry						
Blood sugar (mg/dL)	74–99	215	199	192	172	166
Sodium (mmol/L)	136–145	130	129	128	131	131
Potassium (mmol/L)	3.7–4.8	2.3	2.2	3	3.4	2.9
Chloride (mmol/L)	97–109	87	90	94	91	92
Magnesium (mmol/L)	1.9–2.4	1.6	1.3	3	2.1	1.9
Creatinine (mg/dL)	0.6–1.	1.23	0.97	0.91	0.66	0.39
Albumin (g/dL)	3.5–5.2	4.2	3.8		3.1	
Β-Hydroxybutyric acid (mmol/L)	0.05–0.27	9.95				
Hematology						
Hemoglobin (g/dL)	11.2–15.7	17.3	15.5	13.1		
White blood cells (k/uL)	3.7–10.3	16.41	19.24	11.32		
Platelets (k/uL)	155­–369	541	506	319		
C-reactive protein (mg/dL)	<9	2.4				
Urine ketones (mg/dL)	Not detectable	>/=160				
Glucose (mg/dL)	Not detectable	>/=1000				

Relevant differential diagnoses include euglycemic DKA secondary to the SGLT2i empagliflozin and DKA secondary to acute pancreatitis. Moreover, although rare, empagliflozin-induced pancreatitis was also considered. However, in the setting of a relatively normal lipase and abdominal imaging not suggestive of acute pancreatitis, acute pancreatitis was ruled out.

The patient was diagnosed with euglycemic DKA, likely secondary to empagliflozin, as no other precipitating factor could be identified. However, severe symptomatic hypokalemia despite aggressive repletion limited the use of insulin therapy. The patient was maintained on maintenance normal saline and dextrose 5% with sodium bicarbonate. Empagliflozin was stopped. Intravenous potassium, magnesium, and phosphorus replacements were administered, with regular electrolyte checks. Insulin infusion was avoided of concerns of severe symptomatic hypokalemia and resulting cardiac arrhythmias.

The patient’s renal function improved with intravenous hydration, as did her acidosis and ketonemia. After 24 hours of aggressive potassium repletion, her potassium level raised up to 3.0 mmol/L. As her ketonemia and anion gap acidosis improved with intravenous hydration, she was started on subcutaneous insulin with a small but acceptable decrease in potassium. Electrolyte replacements were switched to oral, empagliflozin was removed from the patient’s list, and the patient was discharged home.

## Discussion

**Figure 1:**
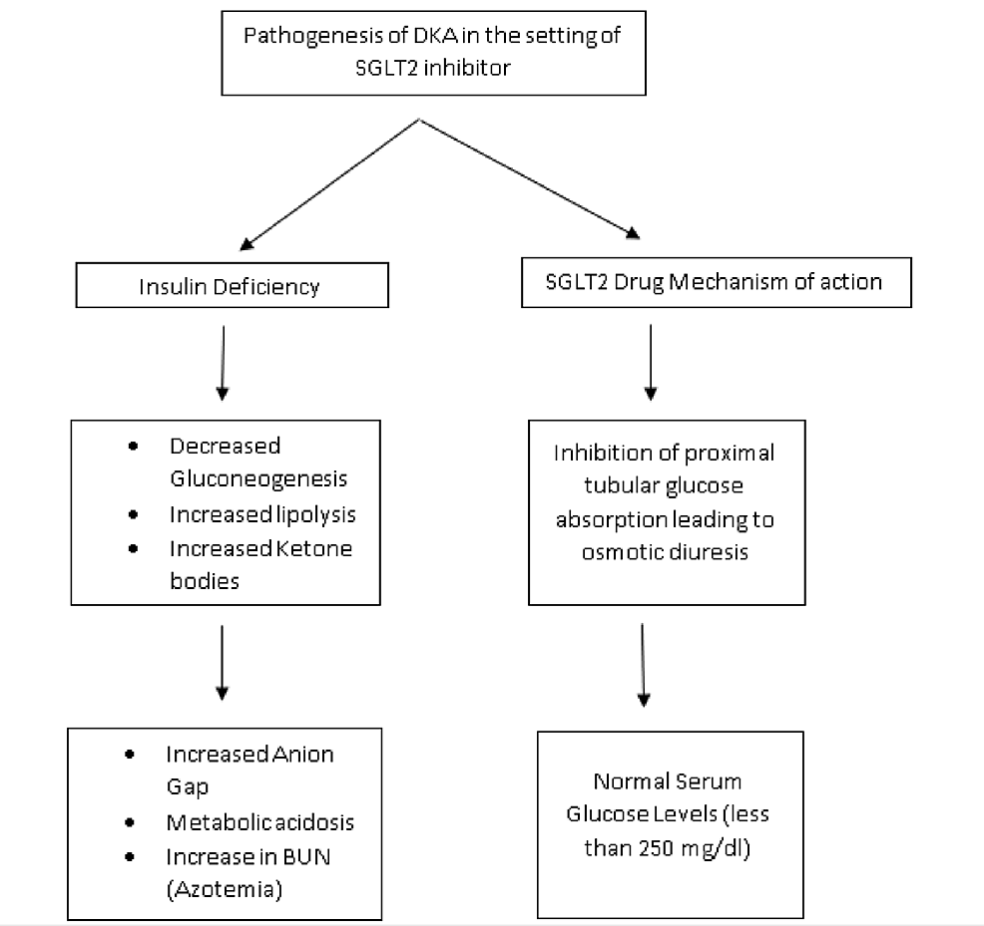
Pathogenesis of diabetic ketoacidosis in the setting of SGLT2 inhibitor use DKA: diabetic ketoacidosis; SGLT2: sodium-glucose cotransporter-2; BUN: blood urea nitrogen

Euglycemic diabetic ketoacidosis (DKA) is defined as a serum glucose level of less than 250 mg/dL (normal range: 140-180 mg/dL in inpatient hospital settings) and a high anion gap (more than 12) with serum bicarbonate of less than 18 mEq/L [4]. The use of SGLT2i in type 2 DM is highly encouraged under ideal circumstances given its beneficial effects especially in reducing heart failure rehospitalizations according to recently published literature [5]. SGLT2i exert their mechanism of action through osmotic diuresis, inducing a euglycemic state. An understanding of the pathophysiology is paramount, with a ketoacidosis state inculcated via increased insulin to glucagon ratio likely induced by starvation, vomiting, pregnancy, dehydration, infection, etc. These triggers lead to a catabolic state with an already depleted glucagon store in the liver, further halting the process of gluconeogenesis and favoring lipolysis with subsequent production of ketones. The tilt of the hormonal balance shifting toward higher concentrations of glucagon, cortisol, and epinephrine further exacerbates this condition and deteriorates the ongoing acidosis. SGLT2i continuously block the proximal tubular reabsorption of glucose; this leads to osmotic diuresis, which balances the glucose levels in the serum, thus leading to a euglycemic state (Figure 1) [6,7].

The rate of occurrence of euglycemic DKA due to SGLT2i is about 0.16-0.76 events per 1000 patient-years in patients with type 2 diabetes [8]. Blau et al. estimate that SGLT2i increase the risk of DKA in patients with type 2 diabetes sevenfold and that risk is increased in patients with beta-cell insufficiency, such as those with type 1 diabetes, explaining why the US Food and Drug Administration does not recommend its use in patients with type 1 DM [9,10]. In terms of management, the highlighting factor is that the use of insulin is remarkably similar to any patient with DKA. Depending on the glucose levels, the concentration of dextrose 5% (5% vs 10%) could be used along with insulin infusion (0.05-0.1 unit/kg/hour). A delay in the institution of an insulin infusion would delay the halting process of ketone bodies and the repletion of normal bicarbonate serum levels. Also, the importance of managing the triggering factor, which in this case was likely pancreatitis, is of utmost value [11]. One of the biggest challenges in managing this patient’s DKA was the delay in insulin drip initiation, which could not be started earlier due to severe hypokalemia in the setting of Gitelman syndrome. This led to a prolonged hospital stay and a delay in the closure of the anion gap metabolic acidosis, so the management had to be adapted to accommodate this peculiar situation. So far in the literature, patients with SGLT2 inhibitor DKA have had a very favorable outcome, such as hyperglycemic DKA due to other reasons; this goes to show that the management plan remains the same. A patient on this medication should be taken off it at least until the acidosis and acute illness are resolved. Careful monitoring of serum potassium and glucose levels is especially important with periodic measurement of serum basic metabolic panel every two to four hours.

## Conclusions

Euglycemic diabetic ketoacidosis (DKA) is a clinical syndrome comprising of normal blood glucose levels, severe metabolic acidosis, and ketonemia. SGLT2i are a novel class of antihyperglycemics and can rarely cause euglycemic DKA. Euglycemic DKA is generally managed on the same principles as conventional DKA, with insulin infusion and intravenous resuscitation. Gitelman syndrome is an inherited renal tubulopathy characterized by hypokalemic metabolic alkalosis, hypomagnesemia, and low urinary calcium excretion. We present a rare clinical encounter of euglycemic DKA due to SGLT2i in a patient with Gitelman syndrome, which turned out to be a management dilemma.
